# Specimens of Morbid Dental Anatomy

**Published:** 1849-07

**Authors:** 


*Specimens of Morbid Dental Anatomy.*—We have recently received from Dr. E. Townsend of Philadelphia, an inferior molaris, with the roots almost entirely united and very greatly enlarged by a deposit of bone. It is a beautiful specimen of exostosis. Accompanying this tooth, is another molar from the same gentleman, in which the whole of the roots are destroyed. The subject from whose mouth it was extracted, applied to a dentist some time before for advice with regard to it. He was, at the time, about seventeen years of age; the nerve was destroyed, and the tooth filled with cotton and tin-foil. The latter had been coming out from time to time, until the tooth, which was a constant source of annoyance to him, was extracted.

We also received at the same time, through the above named gentleman, two teeth from Dr. J. D. White of Philadelphia. Both are inferior molares. In one the pulp is completely ossified. This tooth had been affected, for some time previously to its extraction, with periosteal inflammation. The other is a tooth filled with amalgam, and in which, from its color, copper seems to have been used. We shall take an early opportunity to analyze the filling.

Drs. Townsend and White will accept our thanks for the above specimens.—*Balt. Ed.*

